# Psychometric evaluation of the Chinese version of the burnout syndrome assessment scale in nurses

**DOI:** 10.3389/fpsyg.2024.1309090

**Published:** 2024-02-23

**Authors:** Wenguang Xie, Tingting Lu, Xundong Huang, Chao Zhang, Mahima Choudhary, Ashok Kumar

**Affiliations:** ^1^Department of Nursing, The Second Affiliated Hospital of Nanchang University, Nanchang, China; ^2^College of Nursing, Nanchang University, Nanchang, China; ^3^Department of Nursing, Jinzhou Medical University, Jinzhou, China; ^4^Department of Nursing, College of Xinjiang Uyghur Medicine, Hetian, China; ^5^College of Nursing, All India Institute of Medical Sciences, Jodhpur, India

**Keywords:** nurses, burnout syndrome, factor analysis, psychometric evaluation, reliability, validity

## Abstract

**Objective:**

This study aimed to translate the Burnout Syndrome Assessment Scale (BOSAS) into Chinese and validate its reliability and validity among Chinese emergency department and ICU nurses.

**Methods:**

The scale was translated into Chinese using Brislin’s translation principle. A total of 626 nurses from Jiangxi, Zhejiang, and Fujian provinces in China participated in an online questionnaire survey. The survey included the general information questionnaire for nurses developed by the research team and the Chinese version of the Burnout Syndrome Assessment Scale. Reliability and validity of the Chinese version of the scale were analyzed using SPSS.25 and AMOS.24 software.

**Results:**

The Chinese version of the Burnout Syndrome Assessment Scale consists of a total of 20 items, encompassing two dimensions: personal burnout and job burnout. This structure is consistent with the original English version of the scale. The Chinese version of BOSAS demonstrated high internal consistency, with a Cronbach’s α coefficient of 0.941. Additionally, the scale exhibited good split-half reliability (0.765) and test-retest reliability (0.871). The content validity index (S-CVI) was 0.971, indicating strong content validity. Exploratory factor analysis confirmed the same 2-factor structure as the original scale, and confirmatory factor analysis further validated this structure, with all fit indices indicating appropriateness.

**Conclusion:**

The Burnout Syndrome Assessment Scale has been successfully introduced and its reliability and validity have been verified in Chinese emergency department and ICU nurses.

## 1 Introduction

The shortage of nurses and the imbalance of regional distribution have become a serious global problem. In March of this year, the International Council of Nurses (ICN) declared that the current situation of nurses worldwide is so serious that it should be considered a global health emergency (International Council of Nurses, 2023). According to the report, the global shortage of nurses and midwives was 30.6 million in 2019, and the shortage is expected to worsen in the future (World Health Organization, 2012). According to China’s 2021 census, the population over 60 years old accounts for 18.7% of the total population, and China will enter a stage of accelerated aging in the next few years (State Statistics Bureau, 2021). The increase in the number of elderly people requires an increase in the number of nurses. At the same time, as the population ages, one in six nurses globally will retire within the next decade, exacerbating the nursing shortage (World Health Organization, 2020). It is worth noting that nurse turnover caused by burnout syndrome is one of the important reasons for the shortage of nurses, which further aggravates the shortage of nurses.

Burnout syndrome, initially proposed by Freudenberger in 1974, pertains to the response to prolonged work stress resulting from unfavorable working conditions within the workplace (Güler et al., 1992). Maslach (Gil-Calderón et al., 2021) defines burnout syndrome as a condition characterized by emotional exhaustion and cynicism. It manifests through symptoms at both physical and psychological levels, and can have detrimental effects on the individual’s physical and mental well-being (Choudhary et al., 2022). Studies have shown (Lu et al., 2015; Dall'Ora et al., 2020; Shah et al., 2021) that insufficient staffing of nurses is one of the main causes of nurse burnout syndrome. Compared with other departments, nurses in emergency department and ICU have a higher incidence of burnout syndrome due to factors such as high work intensity, alarm fatigue, and more night shifts (Alharbi et al., 2016; Jiang et al., 2017; Ding et al., 2023). In 16 Asian countries and regions, the incidence of ICU nurse burnout syndrome ranges from 34.6 to 61.5%. Nurses in mainland China have a high degree of job burnout, with an incidence of 61.2% (See et al., 2018). Studies have shown that the COVID-19 pandemic has negatively impacted the health of healthcare workers, further increasing the risk of burnout (Adhikari and Smith, 2023; Bruyneel et al., 2023).

Nurse burnout syndrome has adverse effects on nurses, patients and hospitals. For nurses, burnout syndrome is closely related to the high turnover rate of nurses (Jiang et al., 2017; Shah et al., 2021). Studies have shown that about 46% of nurses will choose to leave the nursing post after experiencing severe job burnout (Boamah and Laschinger, 2016). Other studies have shown that burnout can also lead to a decrease in nurses’ job performance and job satisfaction, which leads to a decrease in nurses’ quality of life (Durkin et al., 2016; Liu et al., 2019). Nurse burnout has been found to be linked with reduced patient safety and an increase in adverse events. These events include patient medication errors, a higher risk of patient infection, and an increased likelihood of patient falls (Liu et al., 2019; Dall'Ora et al., 2020). For hospitals, nurse burnout can lead to an increase in patient complaints and a decrease in revisiting rate, thus causing a huge financial burden to hospitals (Wang et al., 2019).

Therefore, the evaluation and early prevention of nurse burnout syndrome are very important. At present, some scales have been used to assess job burnout, but these tools are universal and have poor specificity for different occupations (Maslach and Leiter, 1996; Kristensen et al., 2005). At present, there is no scale for the assessment of burnout syndrome in nurses in China. Recently, Professor Ashok Kumar’s (Choudhary et al., 2022) team developed the Burnout Syndrome Assessment Scale (BOSAS) to assess burnout syndrome among nurses, which has been validated in India and has shown good reliability and validity. The scale is the first scale specifically used to evaluate nurse burnout syndrome, and it is more targeted than other universal scales. The purpose of this study is to introduce the English version of the Nurse Burnout Syndrome Assessment Scale into China. The aim is to address the issue of a lack of reliable tools for assessing nurse burnout syndrome in China.

## 2 Methods

### 2.1 Design and participants

This multicenter, cross-sectional study was conducted in China from March 2023 to May 2023. A total of 626 nurses were recruited from 3 provinces, namely Jiangxi, Zhejiang, and Fujian, using a convenience sampling method. To ensure the reliability of the research results, 20 nurses participated in each project. In total, there were 20 projects in this scale, with a planned recruitment of 400 nurses. However, considering the non-response or invalid questionnaire of the respondents, a larger sample size may be needed, so 626 nurses were finally recruited in this study (Zhang et al., 2022). Eligible nurses for this study were registered nurses who had a minimum of 1 year of experience in an ICU or emergency department. They were required to have the ability to complete online questionnaires using a smartphone and had to volunteer to participate in the study. Nurses who were practice nurses or standardized training nurses were excluded from the study.

### 2.2 Instruments

#### 2.2.1 Demographic questionnaire for nurses

To investigate the general demographics of emergency department and ICU nurses who participated in this study, a questionnaire was designed after a literature review and extensive discussion by the researchers. The questionnaire included 8 questions, including professional title, daily physical exercise time, and daily contact time with patients and so on.

#### 2.2.2 Burnout syndrome assessment scale

This study utilized the Burnout Syndrome Assessment Scale (BOSAS) developed by Professor Ashok Kumar’s team (Choudhary et al., 2022). The scale comprises 20 items and is categorized into two dimensions: personal burnout (10 items) and occupational burnout (10 items). Each item was assessed using a 5-point Likert scale, ranging from 0 to 4. The scoring criteria were as follows: always = 4, often = 3, sometimes = 2, rarely = 1, and never = 0. Total scores on the scale range from 0 to 80, with higher scores indicating higher levels of burnout. The score ranges for burnout levels are as follows: 0–20 (no burnout), 21–40 (mild burnout), 41–60 (moderate burnout), and 61–80 (severe burnout). The Cronbach’s alpha coefficient for this scale was 0.94, indicating that BOSAS has good internal consistency and can well assess the burnout status of neglect.

### 2.3 Procedures

#### 2.3.1 The Chinese translation process of BOSAS scale

With the authorization of Professor Ashok Kumar, we translated BOSAS scale into Chinese and conducted cross-cultural adjustment. In this study, the translation process follows the internationally popular Brislin’s translation principle (Khalaila, 2013). Initially, two Chinese professors specialized in English translated BOSAS into Chinese. After thorough discussion and negotiation, a Chinese version of the scale was developed. Subsequently, the scale was translated back into English by two foreign professors who had not read the original version. To ensure linguistic conformity, four nursing experts and three psychologists were invited to evaluate the translated scale and provide suggestions for improvement. Based on their expert opinions, a finalized Chinese version of BOSAS was produced. To assess the scale’s clarity and comprehensibility, a preliminary survey was conducted with 30 nurses. The results indicated that the scale’s verbal expressions were easily understood, and it took approximately 3–4 min for participants to complete the scale.

#### 2.3.2 Data collection procedure

In this study, questionnaires were completed among nurses from three provinces in southeast China. During the study period, the researchers of this study traveled to the target hospitals in three provinces for data collection. Nurses from the emergency department and ICU of the hospital were gathered together with the assistance of the director of nursing department of the hospital. The researchers explained the purpose and significance of this study and the method of filling out the questionnaire to nurses on the spot. After the nurses expressed their willingness to participate in this study and signed the informed consent form, the questionnaire was sent to the nurses’ smartphones by the researchers and they were asked to complete the questionnaire and submit it on site. The contents of the online questionnaire were mainly the general demographic information questionnaire of nurses and the Chinese version of BOSAS scale. The online questionnaire was produced by the Questionnaire Star APP in China. When the questionnaire was incompletely filled out, the software would send a text reminder and could not complete the submission operation, which reduced the generation of invalid questionnaires to a certain extent. The software can also export survey data for data analysis, which also avoids errors caused by manual data entry. A total of 700 nurses were recruited from the emergency department and ICU in this study. Fifty of them refused to participate in this study due to busy work. The remaining 650 participants completed the questionnaire online, and all questionnaires were completed anonymously. Finally, 24 invalid questionnaires were eliminated, and 626 valid questionnaires were recovered, with a valid questionnaire rate of 96.31%.

#### 2.3.3 Data analysis procedure

##### 2.3.3.1 Items analysis

Composite scores for each scale were calculated based on the evaluation criteria and ranked in descending order. The relationship between the top 27% (high group) and bottom 27% (low group) was assessed using two independent sample *t*-tests to determine if the scales effectively differentiated between the groups. It was generally accepted that an item was considered sufficiently discriminative if it had a critical factor of 3 or more and a *p*-value less than 0.05. The item scale correlation coefficient and Cronbach’s α coefficient were analyzed to assess whether any items should be omitted from the translation scale.

##### 2.3.3.2 Reliability analysis

To assess the internal consistency of the Chinese version of BOSAS, Cronbach’s alpha coefficient and split-half reliability were utilized. The temporal stability of the Chinese version of BOSAS was evaluated through test-retest reliability. For this purpose, 30 nurses from the emergency department and ICU, who volunteered to participate in the study, were randomly selected and assigned numbers. After 2 weeks of completing the questionnaire, the same 30 nurses were surveyed again using identical questionnaires (Lu et al., 2022). All reliability analysis statistical procedures were conducted using SPSS 25 software.

##### 2.3.3.3 Validity analysis

The validity analysis of the Chinese version of the BOSAS scale involved two main aspects: content validity analysis and structure validity analysis. To assess the content validity, the Delphi method was employed, which involved communication with experts via email. Seven reputable nursing experts from China were invited to individually score each item on the scale, using a rating scale ranging from 1 (not relevant) to 4 (highly relevant). This scoring process aimed to determine the relevance of each item to the scale. The item-level content validity (I-CVI) and scale-level content validity (S-CVI) were then calculated based on the ratings provided by the experts. Specifically, I-CVI was calculated by dividing the number of experts who scored each item with 3 or 4 points by the total number of experts. On the other hand, S-CVI was calculated by taking the mean of the I-CVI values for all 20 items. A threshold of I-CVI ≥ 0.78 and S-CVI ≥ 0.9 was used to determine whether the content validity of the translated scale was good. The scale’s construct validity analysis consisted of two parts: exploratory factor analysis (EFA) and confirmatory factor analysis (CFA). The exploratory factor analysis was performed using SPSS 25 software, while the confirmatory factor analysis was conducted using AMOS 24 software. A total of 626 nurses participated in this study and were randomly divided into two groups of 313 nurses each, using SPSS 25 software. The exploratory factor analysis and confirmatory factor analysis were then carried out sequentially.

### 2.4 Ethical approval

The purpose and significance of the study were explained in detail to each participating emergency department and ICU nurse. All nurses voluntarily participated in this study and signed informed consent, and had the freedom to withdraw from this study at any time during the study. At the same time, this study was approved by the Second Affiliated Hospital of Nanchang University (O-Medical Research Lun Review [2023] No. 32).

## 3 Results

### 3.1 General population characteristics

A total of 626 emergency department and ICU nurses were included in the study, comprising 66 men (10.5%) and 560 women (89.5%). Among the nurses, 62.0% were aged between 25 and 34 years, 60.9% were married, 73.3% held a bachelor’s degree, and 66.8% had been working in their current position for 5 years or more. Additionally, 37.8% of the nurses were currently serving as primary nurses. Regarding physical exercise, 70.1% of the nurses reported not having time for it on a daily basis. Moreover, 52.6% of the nurses stated that they spent over 75% of their working time in direct contact with patients. Further information about the general population can be found in Table 1.

**Table 1:** Frequency distribution of demographic characteristics (*n* = 626).

Factors	Group	*n*	%
Age	18–24	88	14.1
	25–34	388	62.0
	35–44	129	20.6
	≥45	21	3.3
Sex	Male	66	10.5
	Female	560	89.5
Marital status	Unmarried	236	37.7
	Married	381	60.9
	Divorced/widowed	9	1.4
Education level	Technical secondary school	2	0.3
	Junior college education	161	25.7
	Undergraduate education	459	73.3
	Postgraduate education	4	0.7
Working years of current post	1–2	109	17.4
	3–4	99	15.8
	≥5	418	66.8
Professional title	Nurse	154	24.6
	Primary nurse	237	37.8
	Nurse-in-charge	222	35.5
	Deputy director, nurse, and above	13	2.1
Exercise time every day (h)	0	439	70.1
	0–1	161	25.7
	1–2	19	3.1
	>2	7	1.1
Daily contact time with patients	≥75	329	52.6
	About 50%	265	42.3
	≤30%	32	5.1

### 3.2 Item analysis

The critical ratio (CR) was utilized to assess the discrimination of each item in the scale. When CR > 3.0, it indicated good discrimination for all items in the scale. The CR values for the 20 items of the Chinese version of BOSAS ranged from 12.812 to 22.763, which clearly exceeded 3, demonstrating good discrimination for each item. This implies that the Chinese version of BOSAS effectively measures burnout syndrome among different nurses. The correlation coefficients (r) between each item and the total translation score of the scale ranged from 0.545 to 0.779 (*p* < 0.001), indicating a moderate to high correlation between each item and the scale. After removing some items, the Cronbach’s alpha for each item ranged from 0.936 to 0.941, all of which were below the Cronbach’s alpha of 0.941 for the converted scale. Therefore, all 20 items in the original scale were retained in the Chinese version of BOSAS (Table 2).

**Table 2:** Item analysis for Chinese version of the BOSAS.

Item	Critical ratio	Correlation coefficient between item and total score	Cronbach’s Alpha if item deleted
Personal burnout-1	21.058	0.709	0.938
Personal burnout-2	20.383	0.697	0.938
Personal burnout-3	14.572	0.545	0.941
Personal burnout-4	22.763	0.779	0.936
Personal burnout-5	21.249	0.759	0.937
Personal burnout-6	22.225	0.770	0.936
Personal burnout-7	20.121	0.700	0.938
Personal burnout-8	15.977	0.627	0.939
Personal burnout-9	18.925	0.662	0.939
Personal burnout-10	22.528	0.761	0.937
Professional burnout-1	19.981	0.760	0.937
Professional burnout-2	21.330	0.757	0.937
Professional burnout-3	19.334	0.738	0.937
Professional burnout-4	15.338	0.626	0.939
Professional burnout-5	14.898	0.601	0.940
Professional burnout-6	19.246	0.726	0.937
Professional burnout-7	17.658	0.674	0.938
Professional burnout-8	19.268	0.687	0.938
Professional burnout-9	15.602	0.620	0.940
Professional burnout-10	12.812	0.547	0.940

### 3.3 Reliability analysis

The Cronbach’s coefficient of the Chinese version of BOSAS was 0.941, which closely matched the results of the original scale (Choudhary et al., 2022). Each dimension of the scale had a Cronbach’sαvalue ranging from 0.915 to 0.925, indicating high internal consistency. The split-half reliability of the translated scale was 0.765. After a period of 14 days, the questionnaire was administered again to 30 nurses. The correlation analysis revealed a test-retest reliability of 0.871 for the Chinese version of BOSAS. Therefore, the translated scale demonstrated appropriate reliability. Please refer to Table 3 for specific indicators.

**Table 3:** Reliability analysis for Chinese version of the BOSAS.

The scale and its dimension	Cronbach’s Alpha	Split-half reliability	Test-retest reliability
The BOSAS	0.941	0.766	0.871
Personal burnout	0.925		
Professional burnout	0.915		

### 3.4 Validity analysis

#### 3.4.1 Content validity analysis

Seven Chinese nursing experts evaluated the content validity of the Chinese version of BOSAS. The results show that the value range of I-CVI was 0.857–1.000, and the value range of S-CVI was 0.971.

#### 3.4.2 Exploratory factor analysis

**Figure 1:**
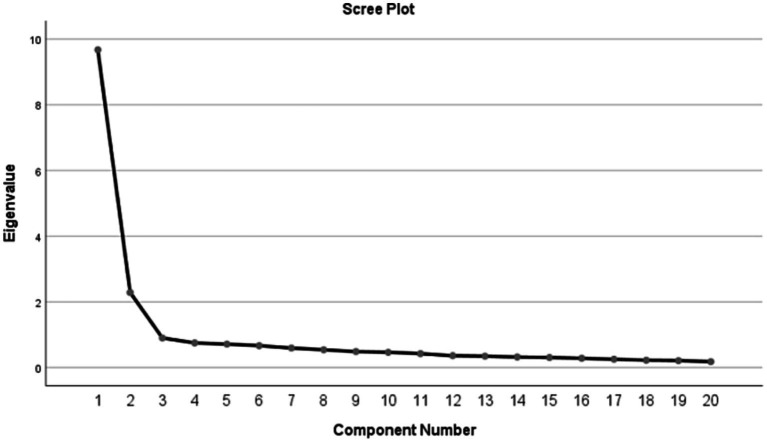
Screen plot of exploratory factor analysis for Chinese version of the BOSAS.

The EFA results showed that KMO = 0.922, and Bartlett’s sphericity test was statistically significant (χ^2^ = 4043.953; *p* < 0.001), indicating the validity of factor analysis. A total of 2 factors with eigenvalues greater than 1 were extracted, explaining 59.795% of the variance in the data. The existence of the 2-factor structure was further demonstrated by the screen plot, it was shown in Figure 1. In addition, the factor loading results are also satisfactory, see Table 4 for specific indicators.

**Table 4:** Factor loadings of exploratory factor analysis for Chinese version of the BOSAS.

Item	Factor 1	Factor 2
Personal burnout-1	0.817	
Personal burnout-2	0.772	
Personal burnout-3	0.659	
Personal burnout-4	0.763	
Personal burnout-5	0.753	
Personal burnout-6	0.700	
Personal burnout-7	0.773	
Personal burnout-8	0.694	
Personal burnout-9	0.663	
Personal burnout-10	0.684	
Professional burnout-1		0.704
Professional burnout-2		0.694
Professional burnout-3		0.689
Professional burnout-4		0.761
Professional burnout-5		0.746
Professional burnout-6		0.734
Professional burnout-7		0.751
Professional burnout-8		0.632
Professional burnout-9		0.691
Professional burnout-10		0.712

#### 3.4.3 Confirmatory factor analysis

**Figure 2:**
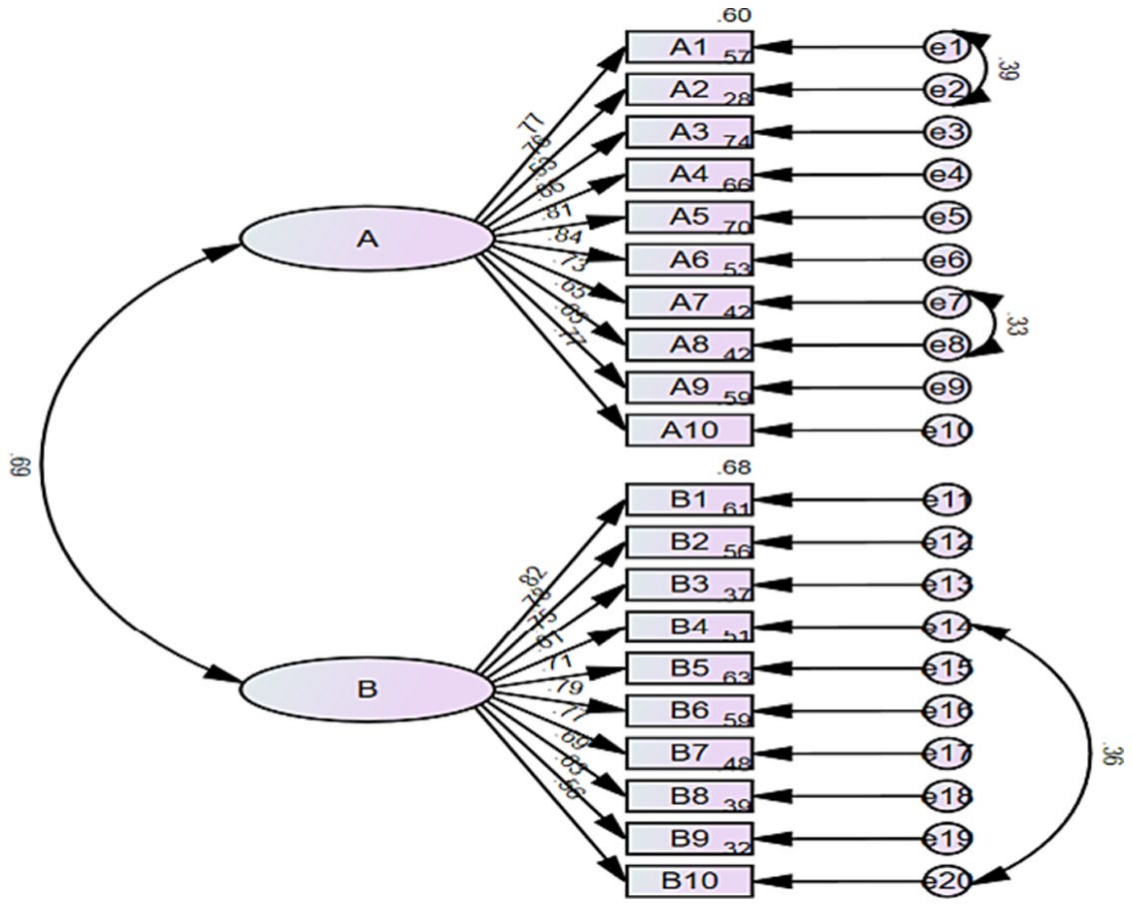
Standardized two-factor model of the Chinese version of BOSAS. **(A)** Personal burnout; **(B)** professional burnout.

The 2-factor structure of the Chinese version of BOSAS was further verified by confirmatory factor analysis, and the results are shown in Figure 2. Based on the correction index, three corrections were made to the initial model, namely e1 and e2, e7 and e8, and e14 and e20. In the end, all indexes showed good results (χ^2^/df = 2.504, CFI = 0.936, TLI = 0.927, IFI = 0.936, RMSEA = 0.069).

## 4 Discussion

The incidence of burnout syndrome is high among nurses, especially in departments with high workloads such as the ICU and emergency department (Jiang et al., 2017; Wang et al., 2019). Therefore, early assessment of burnout syndrome is crucial. Although some scales (Maslach and Leiter, 1996; Kristensen et al., 2005) have been used to assess burnout syndrome in the past, they have certain limitations and may not be fully applicable to nurses. The BOSAS scale (Choudhary et al., 2022) was specifically developed for nurses and can effectively assess burnout syndrome in this group. This study successfully introduced the burnout syndrome assessment scale to China, providing Chinese nursing managers with a reliable tool. The application of this tool enables the assessment of nurses’ burnout syndrome levels and serves as a foundation for nursing managers to develop intervention measures.

The Chinese version of BOSAS consists of 20 items, which are divided into two dimensions: personal burnout and job burnout, following the same structure as the original scale (Choudhary et al., 2022). Currently, BOSAS has only been validated in nurses in India and China, with no relevant literature reports in other countries. The translation process of BOSAS followed Brislin’s double literal translation-back translation model (Khalaila, 2013), including forward translation, back translation, and expert consultation. After inviting 7 Chinese experts to revise the initial translation draft, the Chinese version of BOSAS was finalized. A pre-survey was conducted with 30 nurses, who found the Chinese version of BOSAS to be smooth and easy to understand.

The reliability and validity of the Chinese version of BOSAS were assessed through an online questionnaire survey involving 626 emergency department and ICU nurses in China. Reliability analysis is to check whether the scale is actually measuring its structure (Koo and Li, 2016). In this study, what reflected the reliability of the scale was that all three indicators met the requirements. This suggests that the translated scale is a reliable tool to assess nurse burnout syndrome. The content validity of the translation scale was evaluated by seven nursing experts using Delphi expert letter consultation method. The S-CVI of the translated scale was 0.971, higher than that of the original scale (Choudhary et al., 2022). The potential two-factor structure of BOSAS was determined through EFA, which accounted for 59.795% of the total variance. Additionally, the factor loading of all 20 items exceeded 0.5. The factor structure of the translated scale was consistent with that of the original scale (Choudhary et al., 2022). The underlying factor structure was further verified by confirmatory factor analysis, and all fit indices reached standard values (Shi et al., 2022). In conclusion, the reliability and validity of the Chinese version of the Burnout Syndrome Assessment Scale are satisfactory, making it a reliable tool for assessing nurse burnout syndrome in China. Therefore, it is suitable for widespread use and promotion in the country.

## 5 Limitations

This study has several limitations. Firstly, since all the questionnaires used were self-report, bias is inevitable. Secondly, the sample size of this study primarily consists of participants from the southern provinces of China, while the sample size from the northern provinces is relatively small. Therefore, it is important to conduct further multi-center and large-sample research.

## 6 Conclusion

The reliability and validity of the Chinese version of BOSAS has been verified in Chinese nurses. The scale is a simple and reliable tool, which is suitable for further promotion in China.

## Data availability statement

The data analyzed in this study is subject to the following licenses/restrictions: The authors will provide all original data relevant to this study without reservation. It is available by contacting the corresponding author for valid reasons. Requests to access these datasets should be directed to CZ, ndefy89011@ncu.edu.cn.

## Ethics statement

The studies involving humans were approved by Second Affiliated Hospital of Nanchang University (O-Medical Research Lun Review [2023] No. 32). The studies were conducted in accordance with the local legislation and institutional requirements. The participants provided their written informed consent to participate in this study.

## Author contributions

WX: Methodology, Software, Writing – original draft, Validation, Writing – review & editing. TL: Formal analysis, Methodology, Validation, Writing – review & editing. XH: Data curation, Investigation, Project administration, Writing – review & editing. CZ: Formal analysis, Project administration, Resources, Visualization, Writing – review & editing. MC: Resources, Writing – review & editing. AK: Resources, Writing – review & editing.
